# A pilot study of disparity vergence and near dissociated phoria in convergence insufficiency patients before vs. after vergence therapy

**DOI:** 10.3389/fnhum.2015.00419

**Published:** 2015-07-27

**Authors:** Tara L. Alvarez

**Affiliations:** Department of Biomedical Engineering, New Jersey Institute of TechnologyUniversity Heights, Newark, NJ, USA

**Keywords:** convergence, divergence, fusion initiating component, near dissociated phoria, convergence insufficiency, vergence therapy

## Abstract

**Purpose:** This study examined the relationship between the near dissociated phoria and disparity vergence eye movements. Convergence insufficiency (CI) patients before vergence therapy were compared to: (1) the same patients after vergence therapy; and (2) binocularly normal controls (BNC).

**Methods:** Sixteen subjects were studied—twelve BNC and four with CI. Measurements from the CI subjects were obtained before and after 18 h of vergence eye movement therapy. The near dissociated phoria was measured using the flashed Maddox rod technique. Vergence responses were stimulated from 4° symmetrical disparity vergence step stimuli. The peak velocity of the vergence response and the magnitude of the fusion initiating component (FIC) from an independent component analysis (ICA) were calculated. A linear regression analysis was conducted studying the vergence peak velocity as a function of the near dissociated phoria where the Pearson correlation coefficient was computed.

**Results:** Before vergence therapy, the average with one standard deviation FIC magnitude of convergence responses from CI subjects was 0.29° ± 0.82 and significantly less than the FIC magnitude of 1.85° ± 0.84 for BNC (*p* < 0.02). A paired *t*-test reported that the FIC and near dissociated phoria before vergence therapy for CI subjects significantly increased to 1.49° ± 0.57 (*p* < 0.04) and became less exophoric to 3.5Δ ± 1.9 exo (*p* < 0.02) after vergence therapy. A significant correlation (*r* = 0.87;* p* < 0.01) was observed between the near dissociated phoria and the vergence ratio of convergence peak velocity divided by divergence peak velocity.

**Conclusion:** The results have clinical translational impact in understanding the mechanism by which vergence therapy may be changing the vergence system leading to a sustained reduction in visual symptoms.

## Introduction

Convergence is the inward rotation of the eyes such as when a person changes gaze from looking at a target located far to an object located closer to subject. Divergence is the outward rotation of the eyes (i.e., looking at a target close and then changing gaze to a target located further away from the subject). Vergence oculomotor movements are malleable with training/therapeutic rehabilitation, which is presumably due to changes in the neural substrates that mediate the vergence response. For example, convergence insufficiency (CI) subjects increase their convergence peak velocity when exposed to repetitive vergence training (van Leeuwen et al., 1999; Alvarez et al., 2010; Jainta et al., 2011). Sustained improvement in convergence peak velocity 1 year post therapy is reported for adults with CI (Alvarez et al., 2010). Near point of convergence, positive fusional amplitude and visual symptoms in children with CI studied within a randomized clinical trial also exhibit sustained improvements 1 year post-therapy [Convergence Insufficiency Treatment Trial (CITT) Study Group, 2009]. Such sustained improvements suggest potential changes to the neural substrates that stimulate convergence responses, which may in part be evoked as a result of repetitive vergence therapy (Scheiman et al., 2011).

Early investigations quantitatively measured and modeled vergence oculomotor responses as a pure feedback controlled system (Rashbass and Westheimer, 1961). However, many behavioral and modeling studies now support that a vergence response to a symmetrical stimulus along the midline is composed of two components. The first is named the fusion initiating component (FIC), which mediates the transient portion of the movement and is responsible for the movement’s maximum velocity. The second is named the fusion sustaining component (FSC), which mediates the sustained portion of the movement and is responsible for the movement’s accuracy (Jones, 1980; Hung et al., 1986; Semmlow et al., 1986; Horng et al., 1998; Chen et al., 2010; Lee et al., 2012). One challenge in the study of vergence neural control behavior is that both the FIC and FSC are stimulated during a vergence response. Hence, it is difficult to disentangle the two components in the oculomotor vergence response. To address this challenge, a blind source separation technique known as independent component analysis (ICA) was validated as a tool to perform a “dry dissection” of eye movements and isolate the FIC and FSC subcomponents of vergence (Semmlow and Yuan, 2002; Semmlow et al., 2002, 2007). Although vergence responses typically have a combination of the FIC and the FSC, the type of visual stimulus can produce varying amounts of the FIC and the FSC, especially during the transient portion of the vergence movement (Alvarez et al., 1998, 1999, 2000; Yuan et al., 1999). Step responses have been shown to be driven more from the FIC than from the FSC during the transient portion of the vergence movement, whereas the FSC dominates the late and steady-state portion of the response (Alvarez et al., 1998, 1999, 2000; Semmlow and Yuan, 2002; Semmlow et al., 2007).

Disparity vergence is sometimes referred to as the “fast” portion of vergence (also called voluntary-disparity or phasic vergence) because it rapidly reduces initial disparity vergence error. Disparity vergence is also modified by the near dissociated phoria sometimes referred to as the “slow” portion of vergence. Schor states the “fast” and “slow” portions of vergence interact (Schor, 1979, 1980, 1985, 1988, 2009; Schor and Horner, 1989; Maxwell et al., 2012). Modeling studies support that the near dissociated phoria induces a bias or scalar shift to the vergence system (Saladin, 1986, 1988; Schor, 1988). Alvarez and colleagues studying binocularly normal controls (BNC) report that the phoria correlates to phasic vergence peak velocity (fast vergence)—exophores have slower convergence (take longer to reduce disparity error) compared to the same subject’s divergence movements (Kim et al., 2010). Studies also support that as the phoria is adapted via an exophoric shift, a decrease in convergence peak velocity is observed (Patel et al., 1999; Ying and Zee, 2006; Lee et al., 2009; Kim et al., 2011a,b; Kim and Alvarez, 2012). Within longitudinal studies of vergence therapy, several investigators report that the near dissociated phoria significantly changes after therapeutic rehabilitation and is less exophoric compared to the subject’s original baseline measurement (Cohen and Soden, 1984; Daum, 1984). Yet, no investigation on CI has studied whether the near dissociated phoria was correlated to the peak velocity of disparity vergence. This is important because understanding how the near dissociated phoria may correlate to the vergence peak velocity may yield insight as to why vergence therapy leads to a sustained reduction in a patient’s visual symptoms. In addition, the peak velocity of vergence responses contains a combination of the FIC and FSC. The fast fusional system can be dissected into the FIC and FSC using ICA. This study will also investigate the magnitude of both the FIC and FSC to determine whether vergence therapy is modifying either or both components.

The novelty of this study is to investigate the near dissociated phoria and the FIC of vergence in patients with CI before and after vergence therapy compared to BNC. The primary aims of the study are the following: (1) determine whether the FIC is reduced in CI subjects compared to BNC and improves post vergence therapy; (2) determine whether the near dissociated phoria becomes less exophoric after vergence therapy; and (3) assess whether a correlation exists between the near dissociated phoria and the vergence peak velocity ratio of convergence divided by divergence. This present study will investigate responses stimulated via abrupt changes in disparity using symmetrical convergence step stimuli.

## Materials and Methods

The methodology will be presented in the following subsections: (1) subject attributes; (2) near dissociated phoria and vergence eye movement measurements; (3) validation of ICA for vergence eye movements; and (4) statistical analyses.

### Subject Attributes

Sixteen subjects participated within this study. Twelve BNC subjects with normal binocular vision between 20 and 28 years of age and four subjects diagnosed with CI between 19 and 25 years of age. The CI subjects were all female while six of the BNC were female and six were male.

Normal binocular vision was defined by the subject’s near point of convergence and stereopsis. All BNC had a normal near point of convergence (<7 cm), assessed by measuring with a ruler the distance a high acuity target was perceived as diplopic along the subject’s midline. BNC subjects also had normal stereopsis (≤50 s of arc), assessed by the Randot Stereopsis Test (Bernell Corp., South Bend, IN, USA). All subjects wore their corrected refraction during the experiments. Subjects were excluded if their prescription was greater than 2D for either myopia or hyperopia.

The four CI subjects were diagnosed with CI by an optometrist. CI was diagnosed when the patient’s near point of convergence was greater than 6 cm and failed Sheard’s criterion, which states that the fusional vergence reserve should be at least twice the magnitude of the near dissociated phoria (measured at 40 cm along midline; Cooper et al., 1998). The visual parameters for the CI subjects and BNC are reported in Tables 1, 2, respectively. All these methods were described in detail in a previous investigation by this team (Alvarez et al., 2010). All CI subjects had local stereopsis levels of ≤ 50 s of arc, assessed using the Randot Stereopsis Test. Three of the CI subjects (S1, S2 and S4) did not need refractive correction, whereas subject S3 was myopic (a prescription of −1.25D for the right eye and −2.25D for the left eye). Ten of the twelve BNC did not require refractive correction. One of the BNC had a prescription of −1D for the right and left eye. Another BNC had a prescription of −1.5D for the right eye and −1.25D for the left eye.

**Table 1:** **Clinical parameters from the CI subjects before and after vergence therapy for near point of convergence (NPC), recovery point of convergence (RPC), positive (base out) and negative (base in) fusional range, and dissociated near phoria**.

Parameter	Time relative to vergence therapy	CI Subjects			
		CI1	CI2	CI3	CI4
Near point of convergence (cm)	Before	13.5	11	9	22
	After	7	6	6	11
Recovery point of convergence (cm)	Before	23.5	24.5	11	24
	After	13.5	10	9	13
Positive fusional range (Δ)	Before	20	10	10	18
	After	40	35	45	40
Negative fusional range (Δ)	Before	10	12	16	14
	After	8	10	16	14
Dissociated near phoria (Δ)—is exophoria	Before	−10	−9	−10	−8
	After	−2	−2	−6	−3

**Table 2:** **Clinical parameters from the BNC subjects for near point of convergence (NPC), recovery point of convergence (RPC), positive (base out) and negative (base in) fusional range, and dissociated near phoria**.

Parameter	Subjects											
	S1	S2	S3	S4	S5	S6	S7	S8	S9	S10	S11	S12
Near point of convergence (cm)	6	5	5	4.5	5	6	7	6	4	5	6	5
Recovery point of convergence (cm)	8	6	7	6	9	8	8	9	7	8	7	8
Positive fusional range (Δ)	25	30	20	25	40	45	30	35	30	45	35	25
Negative fusional range (Δ)	10	12	14	10	18	16	16	14	12	25	20	20
Dissociated near phoria (Δ) + is esophoria and—is exophoria	4	4	2	−1	−2	−2	−3	−6	−6	−7	−7	−8

This study was approved by the Institution Review Board (IRB) of the New Jersey Institute of Technology which was in accordance with the Declaration of Helsinki. All subjects signed written informed consent approved by the IRB committee.

### Near Dissociated Phoria and Vergence Eye Movement Measurements

#### Near Dissociated Phoria Measurement

The near dissociated phoria was subjectively measured using a Maddox rod and the Bernell Muscle Imbalance Measure (MIM) card (Bernell Corp., South Bend, IN, USA). The MIM has a resolution of 1Δ and a range of 28Δ exophoria to 28Δ esophoria. The MIM card is calibrated for the right eye; hence, the phoria was measured with the left eye fixating on a medical penlight shown through the MIM card. The target was placed at 40 cm away from the subject’s midline. The visual target equates to an accommodative demand of 2.5 D. The “flashed” Maddox rod procedure used within this study occluded the subject’s right eye for 15 s followed by rapid and brief uncover/cover to assess the red streak placement on the MIM card calibrated grid. Typically three or four flashes were presented where the flashes were repeated until the subject could confidently report on which number the red streak appeared.

#### Eye Movement Instrumentation

Eye movements were recorded using an infrared (λ = 950 nm) limbus tracking system manufactured by Skalar Iris (model 6500, Delft, Netherlands). All of the eye movements were within the linear range of the system (±25°). The system has a high degree of linearity, within 3% between ±25° horizontally (Horng et al., 1998). Digitization of the individual left-eye and right-eye movements were sampled at 200 Hz using a 12-bit digital acquisition hardware card (National Instruments 6024 E series, Austin, TX, USA). Green light emitting diodes (LEDs), 2 mm wide by 25 mm in height with a wavelength of 555 nm, were used for the target step stimuli (Stanley model MU07 part 5101, London, OH, USA). Subjects were situated in a forehead and chin rest assembly to reduce the influence of the vestibular system (Khojasteh and Galiana, 2007). To allow the subject to blink between experimental trials, subjects initiated an experimental trial by pressing a button. Allowing the subject to initiate the experimental trial also reduced the influence of fatigue (Yuan and Semmlow, 2000).

#### Eye Movement Visual Stimuli

Convergence 4° symmetrical step beginning at an initial vergence angle of 2° or 4° were stimulated and recorded. The different initial vergence angles reduced anticipatory movements. No significant difference in peak velocity was observed between movements that began at 2° or 4°. All stimuli were presented after a random delay of 0.5–2.0 s. Divergence 4° steps starting from an initial 8° vergence angle were randomly intermixed with convergence stimuli. Both the randomization of stimulus direction (convergence compared to divergence) and the time when the stimulus was presented ensured that the subject could not predict when the next stimulus would begin. Such a design is important because prediction is reported to influence both the latency and peak velocity of vergence responses (Alvarez et al., 2002, 2005a; Kumar et al., 2002).

#### Repetitive Vergence Therapy Protocol for Convergence Insufficiency Subjects

Repetitive vergence therapy was utilized to presumably provoke changes in the neural substrates that stimulate the vergence oculomotor responses. The CI subjects participated in a total of 18 h of vergence therapy, 6 h at home and 12 h in the laboratory. Home therapy entailed two 10-min sessions (morning and evening) 3 days per week for 6 weeks. Laboratory therapy was composed of 1-h sessions, twice per week for 6 weeks. Within a single day, a subject participated in either laboratory or home therapy but not both. Eye movements were not recorded during the therapy. The laboratory and home therapy consisted of step and ramp stimuli similar to methods used clinically (Griffin, 1988; Scheiman and Wick, 2008).

The step stimuli used within the laboratory vergence therapy were symmetrical convergent 2°, 4° and 6° steps as well as 4° symmetrical divergence steps shown along the subject’s midline using LED targets. Subjects were instructed to track stimuli starting at an initial position of 2° or 4° symmetrical vergence demand for convergence steps, and 8° symmetrical vergence demand for divergence steps. Prior studies have shown that convergence at 2° or 4° were not significantly different within BNC (Alvarez et al., 2005b). Data were acquired for 4 s to ensure adequate time was given to allow the subject to fuse the new target. Subjects performed approximately 25–30 min of repetitive step training per session.

Ramp stimuli used within the laboratory vergence therapy were 1°/s and 2°/s between the range of 2° to 8° symmetrical vergence demand. For the 1°/s ramp, the visual stimulus was presented for 12 s and for the 2°/s ramp, the visual stimulus was presented for 6 s. The ramp stimuli were presented using a haploscope. Two computer screens were used to generate a symmetrical disparity vergence stimulus along the subject’s midline. The stimulus was a green vertical line 2 cm in height and 2 mm in width with a black background. Two partially reflecting mirrors projected the two vertical lines from the computer screens into the subject’s line of sight. The subject performed approximately 25–30 min of repetitive ramp training per session.

#### Eye Movement Analysis

A custom MATLAB™ (Waltham, MA, USA) program was used for all eye movement analyses. Left-eye and right-eye movement data were converted into degrees using the individual calibration data. Calibration for vergence step responses were composed of four sustained positions (1°, 2°, 3°, and 4° inward rotation for each eye). Vergence was calculated as the difference between the left-eye and the right-eye movement to yield a net vergence response. Convergence responses were plotted as positive. Blinks were easily identified based upon manual inspection of the left and right-eye movement response. Responses with blinks at any point during the movement were omitted (up to 4.6% of the data depending upon the subject). Saccadic eye movements were easily identified because of their greater peak velocity compared to vergence. Responses with saccades during the transient portion of the vergence movement were omitted from the analysis because saccades are known to increase the velocities of vergence responses (Zee et al., 1992). To ensure saccades were not present during the transient portion of the response, the left eye and the right eye vergence position and velocity responses were individually inspected. The vergence peak velocity was computed using a two-point central difference algorithm (Bahill et al., 1982).

A prior investigation from our laboratory reported convergence peak velocity was significantly less in CI subjects compared to BNC. No significant difference in divergence peak velocity was observed between the groups. The same study reported convergence peak velocity after vergence therapy significantly increased compared to baseline measurements where significant changes in divergence peak velocity were not observed (Alvarez et al., 2010). Hence, only convergence responses to symmetrical 4° steps were analyzed using ICA to compute the magnitude of the FIC.

### Validation of Independent Component Analysis (ICA) for Vergence Eye Movements

**Figure 1:**
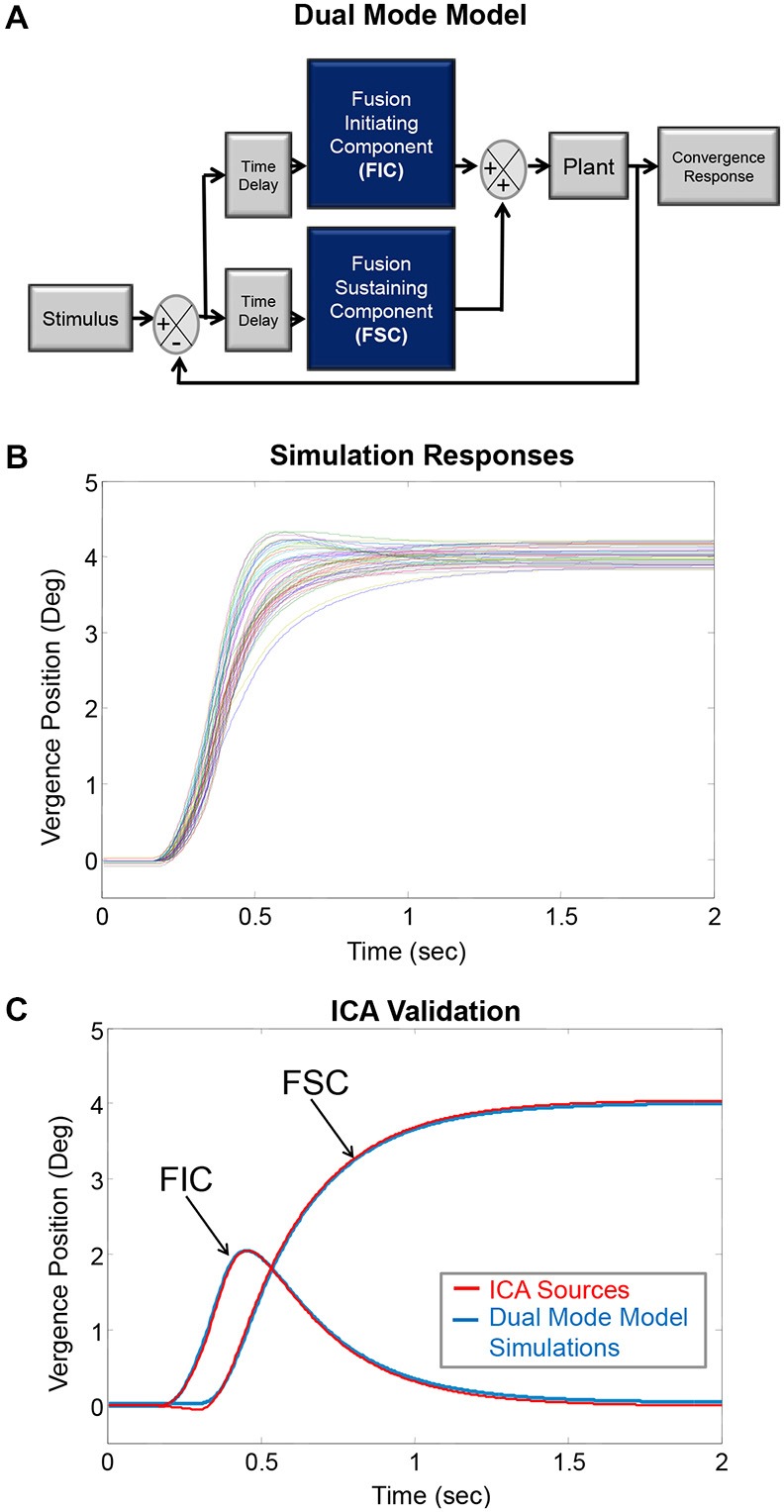
**(A)** The Dual-Mode model describing vergence as preprogrammed with feedback controlled subcomponents termed the fusion initiating component (FIC) and fusion sustaining component (FSC), respectively. These components lead into the plant, which represents mostly the extraocular muscles. **(B)** This model generated simulated convergence responses to 4° symmetrical steps where each colored trace represents a convergence eye movement. These simulated responses are similar to experimental data. **(C)** The simulated responses had known underlying sources. The average FIC and FSC from the known sources shown in plot **(B)** are plotted in blue (labeled using arrows). The ICA algorithm computed two sources plotted in red. The known simulation sources and the ICA sources are similar; validating ICA can be used to dissect experimental convergence responses into their underlying FIC and FSC sources.

In humans, researchers cannot directly record the FIC and FSC convergence signals from the neurons, which innervate the plant (the mechanics of the eye movement system composed mostly as the extraocular muscles) shown in the Dual-Mode model in Figure 1A (Hung et al., 1986; Lee et al., 2012). The use of signal processing is needed to observe the individual subcomponents. Principal component analysis has confirmed that two components describe the majority of variability found in an ensemble of convergence eye movements by plotting a screen plot (eigenvalues as a function of eigenvalue number; Semmlow and Yuan, 2002; Semmlow et al., 2007). Hence, two systems are mostly responsible for the combined convergence response. For example, the FIC and FSC components are both present and combined in convergence oculomotor movements. When a convergence eye movement is recorded, the recording contains a combination of the two components. ICA is a technique that is used to dissect signals that are occurring at the same time (Comon, 1994; Hyvarinen et al., 2001).

ICA solves the mixed source separation problem “blindly” in that it separates the signal sources without making any prior assumptions, such as the timing or information about the shape of the independent source components in the signal. The ICA model is a generative model; it explains how the sources (FIC and FSC) are mixed to generate the signals based on the linear model: *X = As* where *X* and *s* are vectors representing the signals and sources, respectively. The signals (*X*) are the convergence eye movements, and the sources (*s*) are the underlying FIC and FSC. The mixing matrix (*A*) consists of the movement-to-movement variability.

To validate whether ICA could be applied to vergence responses, vergence data were simulated using the Dual Mode model to create responses of known FICs and FSCs using MATLAB™ (Figure 1A). Thirty FIC and thirty FSC subcomponents were randomly generated to form thirty combined responses (Figure 1B). The combined simulated data were entered into the Fast ICA algorithm (Hyvärinen, 1999). Figure 1C shows the average simulated FIC and FSC (solid blue lines) with the corresponding first and second components generated from ICA (solid red lines). The simulation subcomponents and ICA subcomponents were similar; validating that ICA can be used for a dry dissection to study the FIC and FSC subcomponents of vergence. Complete details of the validation of using ICA to study the neural control of vergence were published in prior literature (Semmlow and Yuan, 2002; Castillo et al., 2006; Alvarez et al., 2007; Semmlow et al., 2007, 2012). In addition, ICA has been used in other studies analyzing the size of the pupil while reading, which is part of the near triad used to observe targets located at different spatial depths (Jainta and Baccino, 2010).

### Statistical Analyses

There were three primary types of measurements investigated within this study. The first was the near dissociated phoria. The second was the vergence peak velocity ratio defined as the peak velocity of convergence divided by the peak velocity of divergence. Convergence and divergence responses were stimulated by 4° symmetrical vergence step stimuli. The third was the magnitude of the FIC calculated using ICA of the 4° convergence step responses.

The subject data were stratified into the following three groups: controls with normal binocular vision, CI subjects before vergence therapy, and the same CI subjects after vergence therapy. An un-paired *t*-test determined the following: (1) whether differences were observed between the BNC and CI subjects before vergence therapy; and (2) whether differences were observed with BNC and CI subjects after vergence therapy. A paired *t*-test determined whether the CI subjects exhibited significant changes in the near dissociated phoria and the convergence FIC magnitude by comparing the post vergence therapy measurements to baseline pre-therapy measurements. A linear regression analysis was conducted to study the following: (1) the relationship between the near dissociated phoria and the vergence peak velocity ratio; (2) the relationship between the near dissociated phoria and the convergence peak velocity; and (3) the relationship between positive fusional range and convergence peak velocity. Significance was defined as a *p* < 0.05. Statistics were calculated using NSC2004 (Kaysville, UT, USA). Figures were generated using MATLAB™ and Microsoft™ Excel.

## Results

The near dissociated phoria for the twelve BNC were between 4Δ esophoria to 8Δ exophoria where the average with one standard deviation was 2.6Δ exophoria ± 4.3Δ. The four CI subjects before vergence therapy were between 8Δ exophoria to 10Δ exophoria where the average with one standard deviation was 9.5Δ exophoria ± 0.1Δ. The CI subjects’ near dissociated phoria before vergence therapy were significantly different compared to the BNC using an unpaired *t*-test (*t* = 3.1; *p* < 0.01). A significant reduction in exophoria was observed after vergence therapy for the CI subjects (*t* = 4.2; *p* < 0.02) as assessed using a two tailed paired *t*-test. The near dissociated phoria after vergence therapy for the CI subjects had a range of 6Δ exophoria to 2Δ exophoria where the average and one standard deviation was 3.5Δ exophoria ± 1.9Δ. An unpaired *t*-test, showed that there was no significant difference for the near dissociated phoria between the CI subjects after vergence therapy and the BNC (*t* = 0.4; *p* = 0.7). All clinical measurements before and after vergence therapy are reported within Tables 1, 2 for the CI and BNC subjects, respectively.

The NPC for the BNC subjects was on average with one standard deviation 5.4 ± 0.83 cm. The NPC for the CI subjects before vergence therapy was on average with one standard deviation 13.9 ± 5.7 cm and significantly different from the BNC assessed using an unpaired *t*-test (*t* = 5.4; *p* < 0.0001). After vergence therapy, the NPC for the CI subjects was improved to an average with one standard deviation of 7.5 ± 2.4 cm. A paired *t*-test showed that the NPC significantly changed (*t* = 4.7; *p* < 0.05). While the change in NPC for the CI subjects was significantly improved, the NPC of CI subjects after vergence therapy was still significantly different compared to the BNC using an unpaired* t*-test (*t* = 2.7; *p* = 0.02). However, the difference was substantially lower compared to the baseline values (*p* < 0.0001).

**Figure 2:**
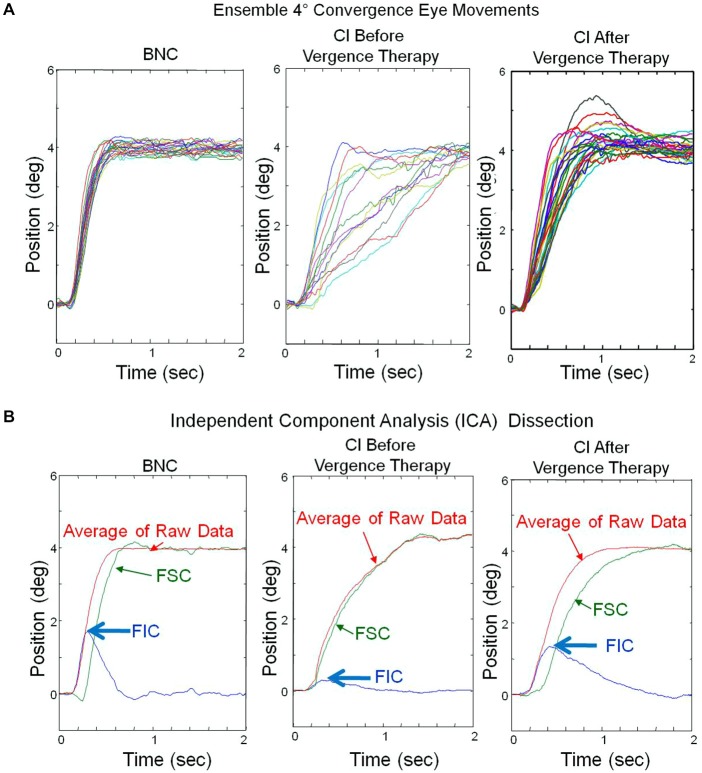
**(A)** Ensemble convergence eye movement responses to 4° symmetrical step stimuli. Each colored trace represents a convergence eye movement. The left plot is responses from a binocularly normal control whereas the middle and right plots are from the same subject with CI before and after vergence therapy, respectively. **(B)** Results from the ICA of the ensemble responses from plots **(A)**. The average of the ensemble eye movements (red), the FIC (blue) and the FSC (green) are plotted. The peak magnitude of the FIC (blue arrow) is reduced in the CI subject before vergence therapy, compared to the BNC, and increases to be more similar to the BNC after vergence therapy.

Ensemble convergence responses to symmetrical 4° step stimuli, located at either 2° or 4° initial convergence demand for one BNC (left), one CI subject before (middle) and the same CI subject after vergence therapy (right) are plotted in Figure 2A. Responses from two different initial convergence demands were investigated because it was unknown whether CI subjects would exhibit significant differences in convergence responses located at 2° or 4° initial vergence demand. The peak velocities at an initial vergence angle of 2° and 4° were not significantly different for either the BNC or the CI subjects (*p* > 0.1) within this study. Hence, these responses were pooled together. Each color trace represents a single convergence eye movement response in Figure 2A. These ensemble vergence responses were entered into the Fast ICA algorithm, which calculated the independent component sources shown in Figure 2B. The FIC and FSC are plotted in blue and green, respectively. The average of the convergence responses are plotted in red. The magnitude of the convergence FIC is depicted by the blue arrows for one BNC and one CI subject before and the same CI subject after vergence therapy in Figure 2B. The FIC of the CI subject before vergence therapy is reduced compared to the BNC, and then the CI subject’s FIC increases to a level closer to the BNC after vergence therapy.

**Figure 3:**
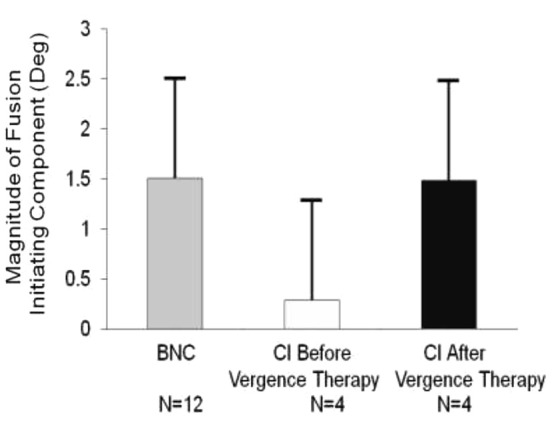
**Group analysis of the average peak magnitude of the convergence FIC for BNC (gray bar), CI subjects before vergence therapy (light gray bar), and the same CI subjects after vergence therapy (black bar)**. Error bars represent one standard deviation. The average convergence FIC for CI subjects before vergence therapy is significantly reduced compared to BNC and then significantly improves post vergence therapy. The number of subjects studied is denoted under each bar.

The magnitude of the convergence FIC was quantified for the twelve BNC and the four CI subjects before and after vergence therapy, as shown in Figure 3, plotted in the gray, light gray and black bars, respectively. The data are plotted as the average plus one standard deviation. An unpaired *t*-test reported significant differences between the convergence FIC magnitude comparing the BNC and CI subjects before vergence therapy (*t* = 2.3; *p* = 0.04). The CI patients had a significantly reduced magnitude of the FIC compared to BNC. A two tailed paired *t*-test reported significant increases in the convergence FIC magnitude after vergence therapy compared to baseline measurements for the CI subjects (*t* = 4.0; *p* = 0.03).

**Figure 4:**
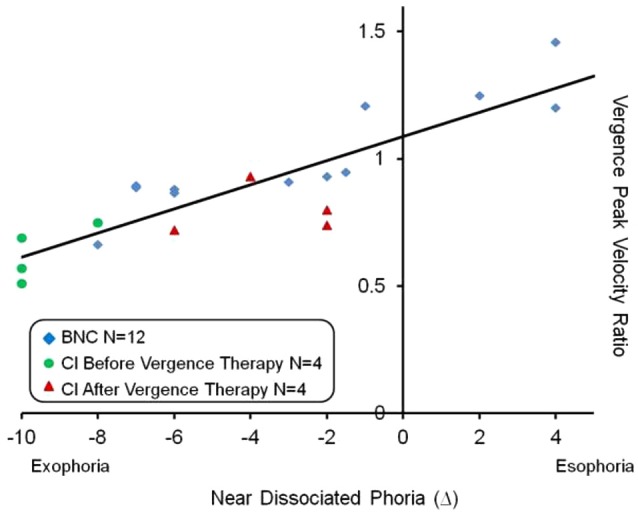
**Linear regression analysis of the vergence peak velocity ratio (average convergence peak velocity divided by average divergence peak velocity) as a function of the near dissociated phoria**. Esophoria is denoted as positive and exophoria is denoted as negative. The BNC, CI subjects before vergence therapy and the same CI subjects after vergence therapy are shown in blue diamonds, green circles and red triangles, respectively. A significant correlation was observed (*p* < 0.01) and the Pearson correlation coefficient was *r* = 0.87.

The vergence peak velocity ratio was defined as the convergence peak velocity divided by the divergence peak velocity. Vergence responses were stimulated from 4° symmetrical vergence step stimuli. A linear regression analysis seen in Figure 4 showed a significant correlation exists between the near dissociated phoria and the vergence peak velocity ratio (*p* < 0.01). The Pearson correlation coefficient was *r* = 0.87. Esophoria was denoted as positive and exophoria was denoted as negative. A vergence peak velocity ratio greater than one means that the convergence responses had a greater peak velocity compared to the divergence responses. Conversely, a vergence peak velocity ratio less than one means that the divergence responses had a greater peak velocity compared to the convergence responses. The data from the BNC, the CI subjects before vergence therapy and the same CI subjects after vergence therapy were denoted in blue diamonds, green circles and red triangles, respectively. Another linear regression analysis was conducted on the convergence peak velocity as a function of near dissociated phoria for all subjects studied. The Pearson correlation coefficient was *r* = 0.58 which was also significant (*p* < 0.01). The vergence peak velocity ratio was not significantly correlated to the positive fusional range (*p* = 0.065).

## Discussion

This study is the first to investigate the magnitude of the FIC and the potential relationship between the near dissociated phoria and the vergence peak velocity ratio in subjects with CI before and after vergence therapy compared to BNC subjects.

### Human Convergence FIC Potential Relation to Primate Neurophysiology Within the Brainstem

Evidence from different laboratories over the last several decades support that the supraocular area (SOA) contains near response cells that modulate their neural activity in response to symmetrical convergent and accommodative stimuli, but not for conjugate (version) or vertical stimuli in primates (Mays and Porter, 1984; Mays et al., 1986; Zhang et al., 1991, 1992; Gamlin, 1999, 2002; Das, 2012). Convergence burst cells in the vicinity of the oculomotor nucleus were described as having the following attributes: a firing pattern that parallels convergence velocity; negligible activity during sustained fixation; spike density that is highly correlated to the convergence movement size; and a burst of activity before the onset of the convergence movement (Mays and Porter, 1984; Judge and Cumming, 1986; Mays et al., 1986). Further investigation of these neural sites revealed that the cells modulated their activity with both vergence and accommodative responses. Hence, these cells were renamed as near response cells within the SOA, located dorsal and lateral to the oculomotor nucleus (Zhang et al., 1991, 1992; Gamlin, 1999, 2002). Recently, investigations of combined saccade-convergence stimuli report that the saccadic burst cells could encode the combined saccadic and convergence movement (Van Horn and Cullen, 2008; Cullen and Van Horn, 2011; King, 2011). However, Van Horn and Cullen (2008) report that the saccadic burst cells did not modulate their activity when presented with a pure symmetrical convergence step stimulus, even though the primate did produce a convergence response.

The ICA decomposition of convergence oculomotor responses from humans shows that the FIC activity is within the transient portion and parallels the movement’s convergence velocity but has negligible contribution to the steady-state portion of the movement (Semmlow and Yuan, 2002; Castillo et al., 2006; Alvarez et al., 2007; Semmlow et al., 2007). The FIC is presumed to be predominantly driven from the neural activity of the near response burst cells described within the SOA because the FIC and burst cells have common attributes. The CI subjects’ pre-vergence therapy measurements compared to post-vergence therapy measurements are of particular interest. The FIC in CI subjects significantly increases after vergence therapy to levels more similar to the magnitude observed in BNC. Results support that the vergence therapy is increasing the magnitude of the FIC. The reduced FIC magnitude in CI subjects compared to BNC is recommended to be investigated in a masked randomized clinical trial. Such a study could determine whether these differences between BNC and those with CI generalize in a larger population and hence may, in part, explain asthenopia in CI patients. This result is important because future therapeutic interventions may look towards methods that further improve the magnitude of the FIC to levels observed within BNC.

### Correlation Between Near Dissociated Phoria and Vergence Peak Velocity Ratio

One very interesting result from this study’s results is the significant correlation between the near dissociated phoria and the vergence peak velocity ratio. The results do not prove causality but do offer more support that the phoria may modulate vergence velocity. One explanation is the phoria may be acting as a spring on the disparity vergence system. For the esophoric subject, the eye’s natural tendency is to deviate inward. Inward rotation would facilitate convergence and impede divergence peak velocity resulting in a vergence peak velocity ratio greater than one, which is exemplified in Figure 4. Conversely, for the exophoric subject, the eye’s natural tendency is to deviate outward which would decrease convergence peak velocity and increase divergence peak velocity resulting in a vergence peak velocity ratio less than one. This behavior is also exemplified in Figure 4. Our laboratory has published a series of different experiments showing that within BNC, the subject’s phoria level can facilitate or impede vergence velocity (Lee et al., 2009; Kim et al., 2010, 2011a,b; Kim and Alvarez, 2012) which is also supported by another independent study (Satgunam et al., 2009). Of particular interest is when a subject’s phoria level changes, so does the vergence peak velocity ratio where the change in both parameters in significantly correlated (Kim and Alvarez, 2012).

Within this present study, the CI subjects had a significant change in their near dissociated phoria level where all four subjects became less exophoric after vergence therapy. Not only did their convergence peak velocity increase but the data in Figure 4 suggests that the phoria may act as an offset for the disparity vergence system. It other words, the near dissociated phoria may be a primary variable to predict the ratio of convergence to divergence peak velocity. Perhaps one of the mechanisms by which vergence therapy leads to a reduction is visual symptoms is the near dissociated phoria is adjusted inward (becomes less exophoric compared to baseline measurement). While this study cannot prove causality, the data support that as a subject’s near dissociated phoria becomes less exophoric, the vergence peak velocity ratio increases. Perhaps as convergence peak velocity increases, the effort to reduce disparity is reduced leading to a reduction in visual symptoms. In addition, if the eyes natural tendency to deviate outward is reduced, perhaps the subject has fewer visual symptoms when doing visual work at near (i.e., reading).

The flashed Maddox Rod technique was chosen because of its ease of use. It is used clinically and specifically has been utilized to measure the dissociated phoria for patients with CI (Brautaset and Jennings, 2005). However, this technique can have limitations because it stimulates accommodation. A technique that stimulates accommodation leads to more variability because some subjects may exert more accommodative demand than others. Hence, the effect observed within this study may be more significant if a phoria measurement that did not stimulate accommodative cues was utilized. Future investigation is warranted to determine whether a stronger correlation exists between vergence velocity and dissociated phoria when the dissociated phoria is measured without accommodative cues.

The range of near dissociated phoria within this current study is between 4Δ esophoria to 8Δ exophoria. Abraham et al. (2015) studied 50 subjects between 19–27 years of age and 50 subjects between 28–35 years of age. These two groups are similar to the ages of those investigated within this present study. The average near dissociated phoria was between 1Δ and 2Δ exophoria with a standard deviation of about 4Δ (Abraham et al., 2015). The 12 subjects studied here have an average phoria of 2.6Δ exophoria with a standard deviation of 4.3Δ. Using an unpaired *t*-test showed that the sample population of BNC studied here was not significantly different compared to the published results that studied 100 subjects (*p* > 0.4).

### Clinical Implications of Long-Term Adaptation Evoked Through Vergence Therapy

Understanding the relationship between the near dissociated phoria and the vergence peak velocity ratio has both basic science and clinical applications. Although the majority of humans perform vergence movements with ease, the binocular dysfunction known as CI is reported to be present within 4.2 to 7.7% of the population (Hokoda, 1985; Scheiman et al., 1996; Porcar and Martinez-Palomera, 1997; Rouse et al., 1998, 1999). CI is an eye co-ordination and alignment problem, which can result in visual symptoms when engaged in reading or performing other close work (Scheiman et al., 2011). In addition, the peak convergence velocity of CI patients is reported to be reduced compared to age-matched controls (van Leeuwen et al., 1999; Alvarez et al., 2010; Thiagarajan et al., 2011). Patients with CI report an array of asthenopic symptoms where patients participate in vergence therapy, also known as vision therapy, to reduce symptoms; however, the underlying neuro-physiological basis for improvement in symptoms in CI patients is unknown (Scheiman et al., 2011). Recent functional magnetic resonance imaging (fMRI) studies are starting to investigate the underlying neuro-physiological basis even though the sample size is small (Alvarez et al., 2014; Jaswal et al., 2014).

The recent randomized clinical trial, the Convergence Insufficiency Treatment Trial (CITT), showed that office-based vergence and accommodative therapy with home reinforcement (OBVAT) was successful in 73% of children. CITT’s results support normal or significantly improved symptoms, near point of convergence, and positive vergence amplitude (Scheiman et al., 2009) that were sustained 1 year post therapy in 88% of patients who participated in OBVAT [Convergence Insufficiency Treatment Trial (CITT) Study Group, 2009]. OBVAT is composed of symmetrical, horizontal convergence movements (Cooper et al., 1998; Scheiman and Wick, 2008; Scheiman et al., 2011) similar to the stimuli used within this study. This present study provides new results of the relationship between the near dissociated phoria and the vergence peak velocity ratio. Perhaps one mechanism by which vergence therapy is leading to a sustained reduction in symptoms is through the change in the near dissociated phoria leading to an increase in the vergence peak velocity ratio.

The techniques used within the present study could also be applied to study other oculomotor and vision dysfunctions. For example, Bucci and colleagues studied vergence insufficiency patients whose symptoms included headache and vertigo before and after orthoptic vergence therapy (Bucci et al., 2004, 2006a,b, 2011; Jainta et al., 2011). The techniques presented here could be used to better understand the mechanisms underlying vergence therapy for those with other visual and vestibular dysfunctions. Other dysfunctions are currently beyond the scope of the present investigation because CI was the only vision dysfunction studied.

### Study Limitations and Future Direction

Future studies are needed to determine whether the results presented here generalize to a larger population. The use of a placebo therapy to serve as a control compared to active therapy should also be considered in future designs to determine the influence of the placebo effect. Future studies may also include BNC subjects where some participate in active and others participate in placebo therapy. Recent research supports that training does significantly (*p* < 0.001) modify vergence kinematics in healthy control subjects (Dysli et al., 2015). Such knowledge is important to learn how malleable the vergence system is in both BNC and patients with binocular dysfunctions.

### Conclusion

Results suggest that when vergence peak velocity is reported, the near dissociated phoria should also be reported because of the positive correlation observed within this study. This investigation is the first to assess the magnitude of the FIC in those with CI before and after vergence therapy compared to BNC. CI subjects have a significantly reduced FIC before vergence therapy compared to BNC which significantly increases post vergence therapy. The near dissociated phoria for CI subjects became significantly less exophoric after vergence therapy compared to each subject’s baseline measurement. As the CI subjects near dissociated phoria became less esophoric, the vergence peak velocity ratio increased in a significantly correlated manner. Future therapeutic interventions may develop methods that concentrate on increasing the magnitude of the convergence FIC to levels observed within BNC as well as modifying the CI subject’s near dissociated phoria to become less exophoric.

## Conflict of Interest Statement

The author declares that the research was conducted in the absence of any commercial or financial relationships that could be construed as a potential conflict of interest.
